# Eastern Equine Encephalitis Virus Diversity in Massachusetts Patients, 1938–2020

**DOI:** 10.4269/ajtmh.23-0047

**Published:** 2023-06-20

**Authors:** Rose M. Langsjoen, Autum Key, Nima Shariatzadeh, Christopher R. Jackson, Faisal Mahmood, Knarik Arkun, Sanda Alexandrescu, Isaac H. Solomon, Anne Piantadosi

**Affiliations:** ^1^ Department of Pathology, Emory University School of Medicine, Atlanta, Georgia;; ^2^ Department of Pathology, Brigham and Women’s Hospital, Harvard Medical School, Boston, Massachusetts;; ^3^ Department of Pathology and Laboratory Medicine, Tufts Medical Center, Boston, Massachusetts;; ^4^ Department of Pathology, Boston Children’s Hospital, Harvard Medical School, Boston, Massachusetts;; ^5^ Division of Infectious Diseases, Department of Medicine, Emory University School of Medicine, Atlanta, Georgia

## Abstract

Eastern equine encephalitis virus (EEEV) is a relatively little-studied alphavirus that can cause devastating viral encephalitis, potentially leading to severe neurological sequelae or death. Although case numbers have historically been low, outbreaks have been increasing in frequency and scale since the 2000 s. It is critical to investigate EEEV evolutionary patterns, especially within human hosts, to understand patterns of emergence, host adaptation, and within-host evolution. To this end, we obtained formalin-fixed paraffin-embedded tissue blocks from discrete brain regions from five contemporary (2004–2020) patients from Massachusetts, confirmed the presence of EEEV RNA by in situ hybridization (ISH) staining, and sequenced viral genomes. We additionally sequenced RNA from scrapings of historical slides made from brain sections of a patient in the first documented EEE outbreak in humans in 1938. ISH staining revealed the presence of RNA in all contemporary samples, and quantification loosely correlated with the proportion of EEEV reads in samples. Consensus EEEV sequences were generated for all six patients, including the sample from 1938; phylogenetic analysis using additional publicly available sequences revealed clustering of each study sample with like sequences from a similar region, whereas an intrahost comparison of consensus sequences between discrete brain regions revealed minimal changes. Intrahost single nucleotide variant (iSNV) analysis of four samples from two patients revealed the presence of tightly compartmentalized, mostly nonsynonymous iSNVs. This study contributes critical primary human EEEV sequences, including a historic sequence as well as novel intrahost evolution findings, contributing substantially to our understanding of the natural history of EEEV infection in humans.

## INTRODUCTION

Eastern equine encephalitis virus (EEEV) is a Togavirus in the genus *Alphavirus* and is one of the most severe arboviral diseases of horses and humans in North America. Like all other viruses in the genus, the ∼11 kb genome of EEEV contains two open reading frames (ORFs) flanked by untranslated regions (UTRs). The first ORF contains the nonstructural gene cassette, composed of nonstructural protein (nsP) genes *nsP1–4*; the second ORF contains the nonstructural gene cassette composed of the capsid, envelope protein (E) E3, E2, 6k/transframe, and E1 genes, and is expressed from a separate subgenomic RNA. The clinical manifestations of EEEV infection include systemic febrile illness followed by neurologic disease, including disorientation, seizures, brain inflammation, and coma. This is often accompanied by abnormal neuroimaging results with lesions in the basal ganglia and cerebral cortex and neutrophilic cerebrospinal fluid (CSF) pleocytosis with elevated protein levels. Eastern equine encephalitis virus was reported to have a 62% mortality rate during outbreaks in the early twentieth century,^1^ although recent reports estimate mortality at 30%, with death rates higher in children.^2^ About half of patients who survive are left with neurologic sequelae, including cognitive deficits and seizure disorders.^3^ Diagnosis is primarily made by IgM detection and detection of neutralizing antibodies in CSF or blood, and supportive treatment includes intravenous immunoglobulins.^4^ However, although there is a commercially available inactivated vaccine for horses, there are currently no FDA-approved antivirals or vaccines against EEEV available for use in humans.

Eastern equine encephalitis virus is endemic predominantly to North America, where it primarily circulates between passerine birds via its enzootic mosquito vector, *Culiseta melanura.*^5^ Mammals, including humans and horses, are considered dead-end epizootic hosts and are infected by either *C. melanura* or other secondary epizootic vectors, such as *Ochlerotatus *sp. and *Aedes *sp.^5^ It is hypothesized that the first observation of EEEV in the United States occurred in the 1800s during an outbreak of encephalitis in horses,^6^ although this remains unconfirmed. The virus was not isolated until it resulted in an outbreak in horses and the first recognized human outbreak of EEE in 1938.^7^ Since then, EEEV has been reported at an average rate of 6–8 cases per year in the United States, where it is endemic mainly along the Atlantic coast, especially in Florida and northeastern states, including Massachusetts, New Hampshire, and New York.^8^ Eastern equine encephalitis virus has also been found in areas around the Great Lakes and Gulf coasts.^4^ Interestingly, whereas cases in the northeastern United States follow a seasonal pattern, with most cases occurring between July and October, Florida exhibits a sustained, year-round transmission cycle.^9^ Phylogenetic analyses reveal that EEEV was introduced multiple times to Vermont, with three sequences from 2011 and 2012 originating from two different parent strains related to Florida strains.^10^ Based on phylogenetic studies,^11^ it is therefore believed that Florida serves as a critical reservoir for EEEV in the United States.

Human EEEV infections have generally been rare, with Massachusetts reporting about 115 cases since the first outbreak in 1938. Outbreaks are small and tend to occur in 10- to 20-year cycles, lasting 2–3 years each.^12^ In 1938, during the first documented EEE outbreak, 38 human cases and 25 fatalities were reported in Massachusetts,^13^ and sporadic outbreaks continued through the 1940s and 1950s. Although cases generally waned thereafter, recognized outbreaks began to increase in frequency and magnitude, starting with an outbreak in Massachusetts in 2004–2006,^14^ followed by another outbreak in 2012. Most recently, a major outbreak occurred in Massachusetts from 2019 to 2020, resulting in 17 cases and 7 deaths.^15^

Although population-level EEEV evolution has been a major focus of previous phylogenetic studies,^10^^,^^11^^,^^16^^,^^17^ the intrahost diversity and evolution of EEEV in human infections is poorly understood, partly due to the paucity of cases and difficulty obtaining viral RNA from the central nervous system (CNS). Most available EEEV sequences have been derived from non-human hosts or cultured isolates. However, one recent study compared EEEV populations from the serum and CSF of a single patient and found multiple intrahost single nucleotide variants (iSNVs) specific to the CSF, and these were enriched in certain genome regions (i.e., the 3′ UTR).^18^ The finding that viral populations in the CNS are comprised of different variants than those in general circulation suggests compartmentalized replication and/or a bottleneck at the blood–brain barrier.

To further understand the diversity of EEEV in human infection, we performed viral sequencing and analysis on formalin-fixed paraffin-embedded (FFPE) tissue from six patients with fatal EEEV infection. Tissue samples were collected from the frontal or temporal lobe, thalamus, midbrain, and spinal cord, and the presence and quantity of EEEV RNA were determined by in situ hybridization (ISH). We generated a consensus EEEV sequence for each sample and performed phylogenetic analysis to evaluate relationships between these and available reference sequences from mosquitos, horses, birds, and other mammals. For four patients, we compared consensus-level EEEV sequence changes between tissue samples, and for two patients we additionally analyzed minority variants between tissue samples by characterizing iSNVs. Critically, our samples include not only contemporary EEEV patients (2004–2020) but also a patient from 1938, allowing us to compare unpassaged virus sequences from human infections over nearly 100 years.

## MATERIALS AND METHODS

### Brain tissue samples and EEEV RNA ISH.

Autopsies with brain examination were performed as previously reported, with broad sampling including frontal/temporal lobe, thalamus, cerebellum, midbrain, and spinal cord.^4^^,^^8^ Specific brain regions were chosen to broadly assess the amount of virus and compare sequences across major anatomic subdivisions within the brain and based on availability across the greatest number of subjects. Clinical data were obtained by review of autopsy reports and electronic medical records. ISH staining for EEEV RNA for one representative slide per tissue per patient was performed using probes V-EEEV-SP (Advanced Cell Diagnostics Cat No. 455728; targeting base pairs 8680-9901 of EEEV/H.sapiens/USA/V105-00210/2005) on the Leica Bond System per manufacturer protocols to produce a brown signal.^19^ Positive control (patient with known EEEV infection) and negative control (patient without EEEV infection) slides were stained with each round of ISH staining. Slides were scanned at 40× using an Aperio Leica Biosystems GT450 scanner (Buffalo Grove, IL), and whole slide images (WSIs) were processed using Python 3.8. Regions with artifacts were manually excluded from analysis by first generating low-magnification copies of the WSIs and annotating the artifacts in red using MS Paint (Microsoft, Redmond, WA). These artifacts included environmental contaminants, glue separation, and scratches on the coverslip. Given the similarity in color between the ISH probe and neuromelanin, the same method was also used to manually exclude the substantia nigra in midbrain sections. The pixel counts of the tissue masks were recorded. A standard color deconvolution method^20^ was applied to the tissue regions of the WSIs, and the number of brown pixels was recorded using a threshold of 50. The total number and average number of brown pixels per unit area of tissue was calculated for each WSI.

### RNA extraction and library construction.

Sequencing was attempted for all tissue samples that had undergone ISH staining for each case. To this end, slide scrapings from historical slides (Case A) and scrolls from contemporary tissue blocks (Cases B–F) underwent RNA extraction using FFPE RNA extraction kits (QUICK-DNA/RNA FFPE mini prep kit, ZYMO RESEARCH, Irvine, CA; E.Z.N.A. FFPE RNA Kit, Omega Bio-tek, Norcross, GA). Extracted nucleic acid underwent heat-labile dsDNase treatment (ArcticZymes, Tromso, Norway). cDNA was made from resulting RNA using random hexamer primers (Fisher/Invitrogen) and SUPERSCRIPT III RT (Fisher/Invitrogen) for first-strand synthesis, and New England Biolabs reagents for second-strand synthesis, without amplification. Sequencing libraries were fragmented and indexed using the Nextera XT DNA Library Prep kit (Illumina, San Diego, CA) with dual indexes and 16 cycles of polymerase chain reaction. Libraries were quantified using the KAPA universal complete kit (Roche, Basel, Switzerland), pooled to equimolar concentration, and sequenced on a MiSeq with paired-end 150-bp reads (Illumina). As a negative control, water was included with each batch of samples starting from DNase. As a positive control, in vitro transcribed External RNA Controls Consortium ERCC spike-ins (NIST) were added to each sample prior to cDNA synthesis. Up to three independent replicate libraries (L1–L3) were constructed from RNA for each tissue sample starting from either the original nucleic acid extraction or DNAse-treated RNA. The number of libraries per sample varied based on consensus coverage and whether additional libraries were needed to cover gaps. Libraries that yielded consensus sequences and an average depth of at least 75× were selected for ultra deep sequencing. Sequencing was unsuccessful from some tissues, including most cerebellum samples, and results are not reported here.

### Data processing and bioinformatics.

Raw Illumina reads were trimmed in BaseSpace and then pre-processed using the ViralNGS pipeline to produce merged, unmapped bam files for each patient. When a single library was sequenced multiple times, reads were merged into a single bam file (Library#; i.e., “L1,” “L2,” “L3”). To obtain patient-specific consensus sequences, all libraries from the same patient were combined into one bam file using the merge_and_reheader app in ViralNGS (“all” libraries); to obtain tissue-specific sequences, all libraries from a tissue were merged into one bam file (Library-Merged, “LM” libraries). Resulting bam files were mapped to EEEV reference genome NC_003899, and consensus sequences were generated using the assembly_referencebased app in ViralNGS. These preliminary consensus sequences were submitted to NCBI BLAST^21^ to identify the closest publicly available EEEV sequence, and then this process was repeated for all individual libraries and merged LM libraries using the following references: Genbank no. NC_003899.1 for Patient A sequences; Genbank no. KX029246.1 for Patient B and C sequences; Genbank no. KX029316.1 for Patient D sequences; and Genbank no. MT782294.1 for Patient E and F sequences.

### Consensus analysis and phylogenetics.

Consensus sequences generated from merged LM files were inspected and checked against consensus sequences of individual libraries (L#) and patient-wide libraries (all). Any positions with different nucleotides reported between libraries were verified by visualization of mapped reads and parsed pileup files using bam-readcount v1.0.1.^22^ Any nucleotide changes that did not match visual read inspection and/or reported nucleotide distributions in pileup file, were at positions with aggregate depth < 5 in the LM mapped bam file, or were within 50 nucleotides of a poly-N stretch were corrected to “N”s or degenerate nucleotide codes. This was to ensure the integrity of consensus reporting and conservative estimation of within-host consensus-level changes. The corrected consensus sequences were then used to compare within-host changes between consensus sequences. This comparison only included positions where at least two tissues from a single patient had clear “A,” “T(U),” “C,” or “G” nucleotides called; samples where positions had “N”s or degenerate nucleotides were excluded. A total of 432 reference full-length EEEV genome sequences were downloaded from NCBI virus (https://www.ncbi.nlm.nih.gov/labs/virus/vssi/#/) along with metadata including accession number, Geo Location, Host, Collection Date, and Genbank title. These were further filtered by availability of all metadata and genome completion (> 99%), to a total of 424 full-length sequences. Sequences were aligned using MAFFT v7.487^23^ using –auto setting and UTR sequences trimmed in Geneious (https://www.geneious.com), trimmed sequences realigned in MAFFT, and then identical sequences removed for a total of 240 sequences. Complete sequence and metadata lists are available in Supplemental Table 1. Phylogenetic analysis was performed using IQTree v1.6.12 for Linux^24^ using -bb 1000 -m MFP settings. Briefly, we used model finder^25^ to identify the best model, followed by ultrafast bootstrap to obtain branch supports.^26^ A maximum-likelihood tree was constructed using the GTR+F+I+G$model and was visualized using interactive Tree of Life v 6.5.8.^27^ Alignments were manually inspected in Genious to evaluate mutations potentially associated with human infection, versus mosquito or other mammal.

### iSNV calling.

To identify iSNVs, reads from individual libraries were trimmed, quality filtered, length filtered, and deduplicated using fastp^28^ v 0.23.2 (-D -A -l 25). Reads were aligned to the patient’s consensus sequence using bowtie2^29^ v 2.4.4 (–local -L 25 -N 1 –gbar 15 –rdg 6,1 –rfg 6,1 –score-min G,30,15) and converted to mapped bam file using samtools^30^ v 1.13 (samtools view -@ $threads -bu -F 4 $DirRoot’_bwt2.sam’ | samtools sort -@ $threads - o $DirRoot’_bwt2.bam’). iSNVs were called using V-Phaser2 v2.0 (vphaser2 -i $DirRoot’_bwt2.bam’ -o ./$Dir/$Root’_vphaser2/’ -ps 100 -ig 25 -dt 0 -a 0.001). iSNVs identified by V-Phaser2 were filtered as follows: 1) 1NT-2NT indels and polyN insertions were removed for each library; 2) iSNVs present in at least two replicate libraries were identified, and allele frequencies were extracted from V-Phaser2 output for the respective combined library; and 3) iSNVs present at less than 1% allele frequency were removed. Each remaining iSNV was manually inspected in merged library mapped reads file using Hudson Tablet viewer,^31^ and spurious iSNVs were removed. Spurious iSNVs were defined as any combination of the following: 1) occurring at only one position across multiple reads, 2) occurring only as a motif of mismatches near the read’s end, and 3) occurring in only one direction. The final iSNV list was annotated using custom annotators, and then nucleotides were indexed to EEEV NC_003899.

## RESULTS

### Clinical and pathological findings were similar between patients.

We investigated EEEV sequence diversity using samples from six patients in Massachusetts between 1938 and 2020 (Table 1). Patient A was a 4-month-old male with no known immune compromise who was one of the first EEE patients described by Farber et al.^1^ Patient B, a 13-year-old male, and Patient C, a 5-year-old female, were infected in 2004 and 2005, respectively; both of these patients were young and immunocompetent, although Patient C had a history of seizures prior to infection.^8^ Patient D was a 63-year-old woman, immunocompromised as a result of rituximab therapy for follicular lymphoma, who was infected in 2012.^4^ Patient E was a 59-year-old woman with a history of large granular lymphocytic leukemia, not currently on treatment, who was infected during the 2019 outbreak. Patient F was a 61-year-old woman with breast cancer treated with trastuzumab, docetaxel, and capecitabine, Lyme disease, and arthritis, who was infected in 2020. All of these patients were from Massachusetts and shared similar clinical presentations (i.e., febrile illness followed by neurological signs and symptoms, most commonly seizures/convulsions).

**Table 1:** Demographic, clinical, and autopsy data for eastern equine encephalitis patients

Data	Patient A	Patient B	Patient C
Date infected	1938	2004	2005
Location infected	Massachusetts	Massachusetts	Massachusetts
Age	4 months	13 years	5 years
Sex	Male	Male	Female
Immune status	Immunocompetent	Immunocompetent	Immunocompetent
Preexisting conditions	None	None	History of seizures (2 months to 2 years)
EEE presentation	Fever, convulsions, coma, cyanosis, stiff neck, bulging fontanel	Seizures	Febrile illness, seizures, mental confusion, agitation
Serological status	Unknown	Unknown	Unknown
Treatment	Phenobarbital, sulfanilamide	Acyclovir, anti-epileptic drugs, dexamethasone, mannitol	Vancomycin, ceftriaxone, acyclovir, anti-epileptic drugs
Duration of illness	11 days	4 days	9 days
Autopsy findings			
Diffuse edema	Yes	Yes	Yes
Herniation	No	Yes	Yes
Duret hemorrhage	No	No	No
Inflammatory infiltrates	Yes	Yes	Yes
Vasculitis	No	Yes	No
Fibrinoid necrosis	No	Yes	Yes
Microglial activation/nodules	Yes	Yes	Yes
Necrosis	Yes	No	No
Thrombosis	Yes	Yes	No
Microinfarcts	No	No	Yes
Hypoxic-Ischemic changes	No	No	Yes
Reference	Farber et al.^1^	Silverman et al.^8^	Silverman et al.^8^

	Patient D	Patient E	Patient F
Date infected	2012	2019	2020
Location infected	Massachusetts, USA	Massachusetts, USA	Massachusetts, USA
Age	63 years	59 years	61 years
Sex	Female	Female	Female
Immune status	Compromised (rituximab)	Immunocompetent	Compromised (trastuzumab, docetaxel, capecitabine)
Preexisting conditions	Follicular lymphoma	Large granular lymphocytic leukemia	Breast cancer, Lyme disease, arthritis
EEE presentation	Febrile illness and mild neurological deficits followed by seizures	Febrile illness, neck stiffness, worsening altered mental status, status epilepticus	Fever, altered mental status, neck stiffness
Serological status	Negative serum and CSF EEE IgM	CSF and serum positive EEE IgM	CSF positive IgM and IgG
Treatment	Vancomycin, ceftriaxone, ampicillin, acyclovir, AmBisome, levetiracetam	Broad spectrum antibiotics and antivirals, Propofol, Ativan, Keppra, Lasix and Fosphenytoin	Supportive measures
Duration of illness	16 days	40 days	11 days
Autopsy findings			
Diffuse edema	Yes	Yes	Yes
Herniation	No	Yes	Yes
Duret hemorrhage	Yes	Yes	Yes
Inflammatory infiltrates	Yes	Yes	Yes
Vasculitis	No	No	No
Fibrinoid necrosis	No	No	No
Microglial activation/nodules	Yes	Yes	Yes
Necrosis	No	Yes	Yes
Thrombosis	No	No	No
Microinfarcts	No	No	No
Hypoxic-ischemic changes	No	Yes	Yes
Reference	Solomon et al.^4^	–	–

CSF = cerebrospinal fluid; EEE = eastern equine encephalitis.

**Figure 1.**
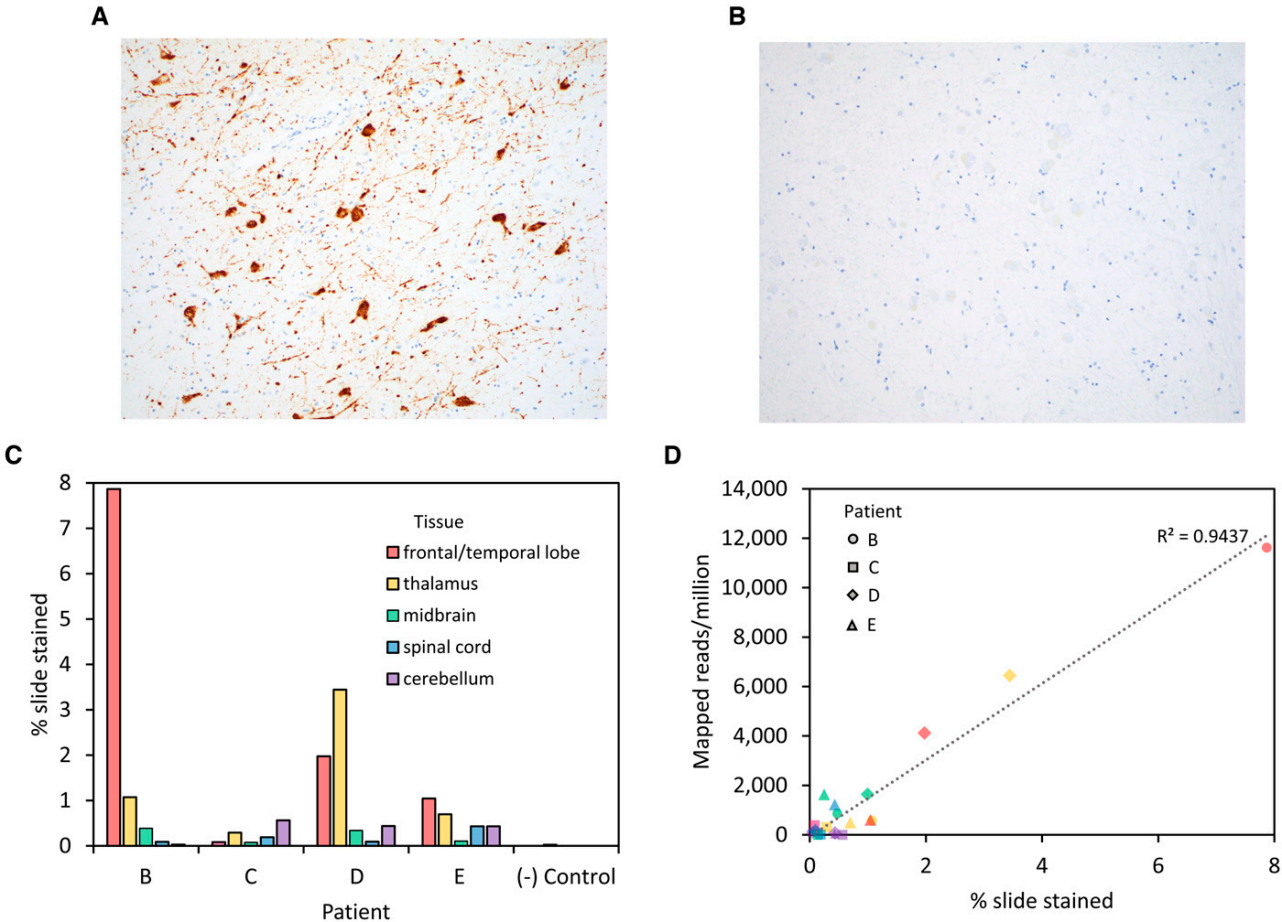
Eastern equine encephalitis RNA in situ hybridization assay and correlation to sequencing results. Eastern equine encephalitis virus (EEEV) RNA in situ hybridization staining of a thalamus section from Patient B shows strong staining of neuronal cell bodies and processes (brown pigment) (**A**), which is absent in thalamus sections from a negative control patient that was not infected with EEEV (**B**). Slides were scanned at 40×, and the percentage of the slide stained was calculated as the total number of brown pixels divided by the total number of tissue pixels on the slide (counterstained blue with hematoxylin) (**C**); results are shown for each section and each individual patient. Staining quantification was compared with results from sequencing a single library from each sample, with shapes representing patients B, C, D, and E and colored by tissue as in panel **C** (**D**). Images in **A** and **B** were taken with 20× objective.

Key postmortem neuropathological findings were also similar between patients, including diffuse edema, perivascular and parenchymal inflammatory infiltrates, necrosis, and microglial activation in all cases (Table 1). Herniation and/or Duret hemorrhages were also common, as were microinfarcts and hypoxic-ischemic changes. We confirmed the presence of EEEV RNA in FFPE slides from Patients B–F by ISH using the RNAScope platform (22 total slides, 2–5 unique slides per autopsy case). Positive staining was qualitatively observed in all sections except spinal cord from Patients B and D and was present in cell bodies and processes, morphologically consistent with neurons (Figure 1A) and was not present in negative control samples (Figure 1B). The amount of staining, quantified from whole slide scanned images by the percentage of the slide with brown pixels, varied between 0.03% and 7.9% across patients and brain regions (Figure 1C). The thalamus and frontal lobe samples had the highest percentage of tissue stained for all patients except Patient C, in whom temporal lobe rather than frontal lobe was analyzed due to sample availability, and cerebellum had the highest percentage of tissue stained. These findings likely indicate higher viral replication in these regions, suggesting a potential within-brain tropism for thalamus and frontal lobe. The level of staining for each sample loosely correlated with the number of EEEV reads per million total reads obtained by metagenomic sequencing in a single library (Figure 1D), indicating that EEEV ISH could be useful in identifying FFPE blocks with the greatest amount of virus for downstream sequencing analyses. In general, all tissues with positive staining had EEEV sequence reads detectable at a minimum sequencing depth of 1,500,000 total reads (Supplemental Figure 1).

### EEEV genome sequences were obtained from most samples.

We obtained complete or near-complete (> 90% coverage) EEEV genome sequences from most tissue samples in this study, including: the frontal lobe, thalamus, midbrain, and spinal cord from Patient B; the thalamus and temporal lobe from Patient C; the frontal lobe, thalamus, and midbrain from Patient D; the thalamus, midbrain, and frontal lobe from patient E; and the hippocampus from Patient F (Table 2). Remarkably, we also assembled about 75% of the EEEV genome sequence from scrapings of 84-year-old slides made from one of the first confirmed human EEEV infections in the United States in 1938 (Patient A). The only other temporospatially similar sequences available are from virus originating from infected horses, isolated between 1933 and 1935 with unknown passage history. Currently, the oldest human isolate is the Decuir strain (GenBank accession KU059747), which was originally isolated from a patient in Louisiana in 1947 and has been passaged several times in various cell types and suckling mouse brain (BioSamples accession SAMN04076100).

**Table 2:** Metagenomic sequencing results and consensus sequence construction for EEEV-positive brain tissue

Patient	Tissue	Total	Mapped	Bases	Ns	Coverage (%)	Depth
A	Brain-all	1.3E+07	820	9,114	2,718	78	4
B	Frontal lobe	5.3E+07	183,772	11,626	0	100.0	1,235
B	Thalamus	1E+07	3,372	10,648	1,218	91.6	26
B	Midbrain	2.7E+07	11,112	11,522	102	99.1	75
B	Spinal cord	3.4E+07	2,447	10,756	943	92.5	17
B	Brain-all	1.3E+08	200,903	11,626	0	100.0	1,355
C	Thalamus	4.1E+07	4,984	11,503	117	98.9	27
C	Temporal lobe	4.7E+07	2,205	10,538	1,114	90.6	12
C	Brain-all	9.8E+07	7,191	11,512	108	99.1	39
D	Frontal lobe	4E+07	37,431	11,627	0	100.0	244
D	Thalamus	4.6E+07	72,235	11,625	0	100.0	476
D	Midbrain	1.6E+07	7,900	11,622	0	100.0	55
D	Spinal cord	1.3E+07	442	7,911	3,865	68.0	2
D	Brain-all	1.5E+08	118,953	11,627	0	100.0	785
E	Thalamus	2.7E+07	1,777	11,642	140	99.5	6
E	Midbrain	3.3E+07	1,131	11,618	232	99.3	4
E	Frontal lobe	2.1E+07	1,356	11,493	473	98.2	4
E	Spinal cord	2.3E+07	268	9,193	3,687	78.6	1
E	Brain-all	1E+08	8,656	11,645	41	99.5	61
F	Hippocampus	3.2E+07	1,219	11,602	0	99.1	13

Bases = bases covered; Coverage = percent reference genome covered; Depth = average nucleotide sequencing depth; EEEV = eastern equine encephalitis virus; Mapped = eastern equine encephalitis–mapped reads; Ns = number of Ns present in consensus sequence; Total = total reads.

### Phylogeny constructed using novel primary EEEV sequences from humans shows no distinct human clades or clustering.

To place these EEEV sequences from humans in the larger context of EEEV diversity from mosquitos and non-human hosts, we aligned our sequences with 240 publicly available complete EEEV sequences from a variety of hosts and temporospatial origins (Supplemental Figure 2).

**Figure 2.**
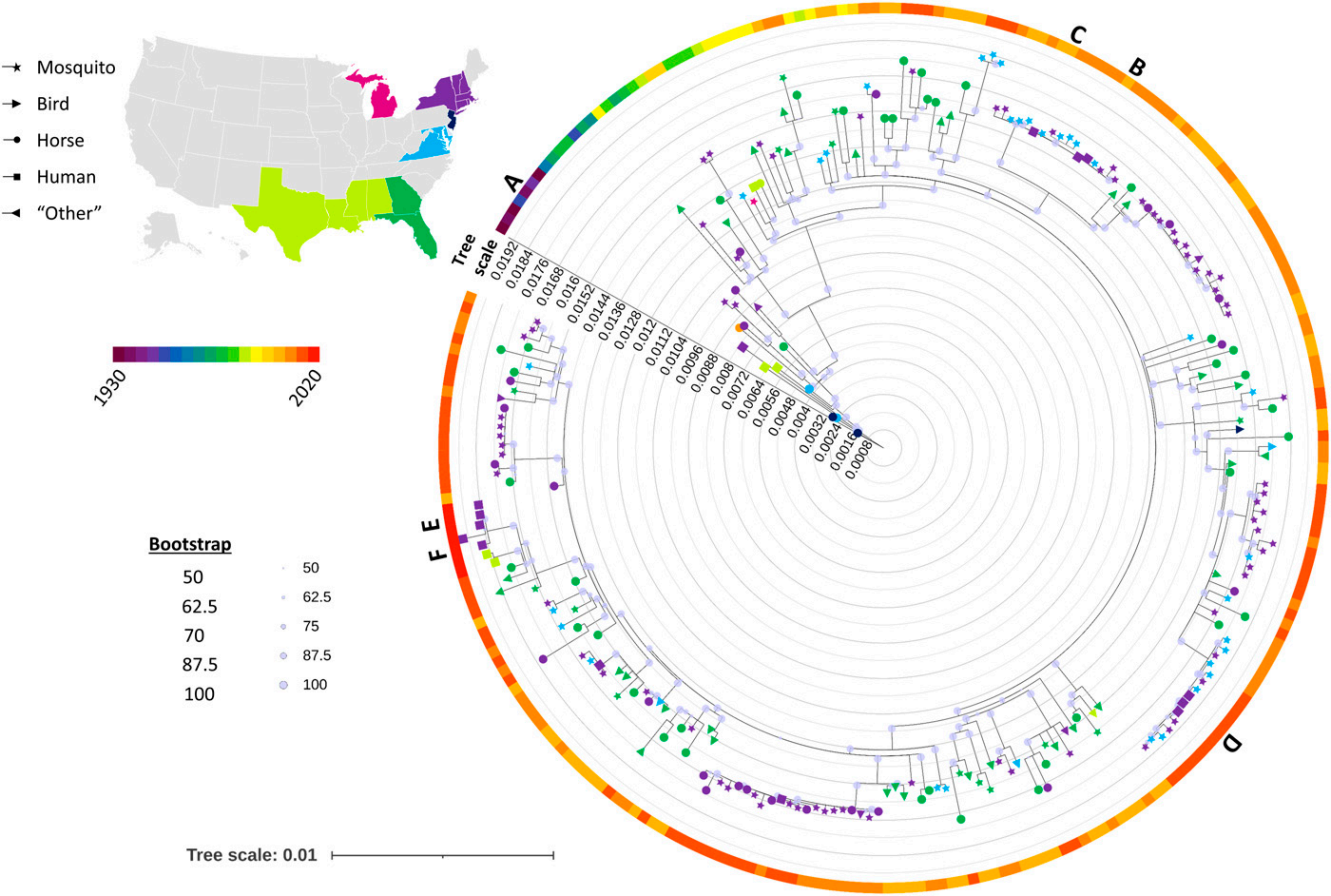
Maximum likelihood phylogenetic tree of historic and contemporary Eastern equine encephalitis virus (EEEV) sequences. Temporospatially diverse full EEEV sequences derived from a variety of hosts (icon shape) were used to construct a maximum-likelihood phylogenetic tree. Tree scale represents genetic distance. Icons are colored by geographic origin, and the surrounding color bar indicates year of the sample. Letters indicate novel human sequences derived from the present study.

Each human sequence clustered with contemporaneous sequences from similar geographic areas, except for Patients E and F, which we attribute to the lack of available sequences from Massachusetts during the 2019 outbreak (Figure 2). Instead, the sequences from Patients E and F clustered with the only available contemporaneous sequences, from a human in Alabama and mosquito, horse, and bird samples from Florida. We compared each human EEEV sequence to its most closely related mosquito and horse EEEV sequences and did not identify any mutations that seemed unique to human infection. One mutation was present in Patient D but nearly no other samples: nsP2 T633N. Although this is a nonsynonymous change in the nsP2 protease protein, the function is currently unknown.

More broadly, sequences clustered by time but not host (Figure 2), as in previous studies. Florida-derived and Northeast-derived samples mostly clustered together, but clades of each intermingled across the tree and Northeast sequences were nearly always nested within clusters of Florida, consistent with the source-sink hypothesis for EEEV maintenance in the United States.^10^^,^^11^^,^^16^ This pattern does not appear to hold for pre-1970 sequences, including our Patient A sample from 1938, likely due to low sampling/availability of sequences from this time.

### Minimal within-host EEEV diversity was observed between brain tissues.

For Patients B, C, D, and E, we recovered complete (> 95%) or near-complete (> 75%) consensus EEEV sequences from multiple distinct brain compartments, including: frontal lobe, thalamus, midbrain, spinal cord, and temporal lobe. This provided a unique opportunity to analyze within-host EEEV diversity in discrete brain regions. No consensus-level changes were observed for Patients B, C, or E. Only two consensus-level changes were observed between distinct brain regions for Patient D (Table 3). One was a synonymous change that occurred in the nsP2 protease gene of virus from the midbrain (nucleotide 3208, at a depth of 8X), which would likely bear no functional consequence for protein function. The other was a nonsynonymous change that occurred in the E1 envelope protein of virus from the spinal cord resulting in a Leucine→Phenylalanine change at residue 162 (nucleotide 10481, at a depth of 10X); although the E1 protein mediates fusion of viral and host membrane, it is unclear what the functional consequences of this change would be.

**Table 3:** Within-host consensus-level changes in EEEV sequences between distinct brain regions

Patient	NT*	RefNT	AltNT	Region	Substitution	ΔAA	Tissue
D	3208	C	T	nsP2	Synonymous	T581T	Midbrain
D	10481	C	T	E1	Nonsynonymous	L162F	Spinal cord

ΔAA = amino acid change; AltNT = alternate allele observed in specific tissue; EEEV = eastern equine encephalitis virusl RefNT = allele in patient-wide consensus genome.

* Indexed to NC_003899.

### iSNV analysis revealed differences in minority variant composition between brain compartments.

**Figure 3.**
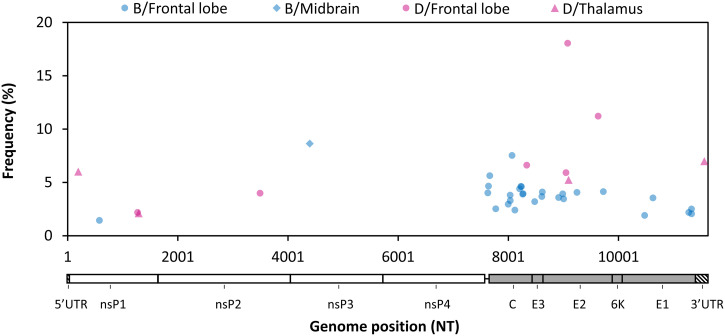
Eastern equine encephalitis virus intrahost single nucleotide variant (iSNV) distribution across different brain compartments. iSNVs that passed our rigorous filtering scheme are plotted with frequency (y-axis) against genome position (x-axis). Each point represents a single iSNV: blue = Patient B, pink = Patient D, and shape indicates tissue type.

To characterize minority variant populations, we performed iSNV analysis of two independent libraries from samples that had a minimum average depth of 75× (two samples each from Patients B and D; Supplemental Table 3), using rigorous filtering to minimize spurious calls. We identified a total of 36 unique iSNVs (Figure 3; full list available in Supplemental Table 4): 28 were present in the frontal lobe of Patient B, 1 was present in the midbrain of Patient B, 6 were present in the frontal lobe of Patient D, and 4 were present in the thalamus of Patient D.

Most iSNVs were concentrated in the structural cassette in the capsid and E2 regions (Figure 3). This was particularly true of the frontal lobe samples, where iSNVs in the structural cassette were disproportionately represented compared with the relative gene sizes. Of the 32 iSNVs that occurred in a coding region, eight were synonymous and 24 were nonsynonymous. Very few iSNVs overlapped either between patients or within patients; only two iSNVs were present in more than one tissue, and both occurred in Patient D. One was an Adenine to Guanine mutation at nucleotide position 9095, resulting in a Serine→Glycine mutation in E2 residue 179, present in the frontal lobe at 18% frequency and the thalamus at 5% frequency. The other iSNV was a three-nucleotide deletion at nucleotide 1223 in nsP1 with allele frequency near 2% in both compartments. The function of this deletion is currently unknown.

## DISCUSSION

We successfully detected EEEV RNA in FFPE sections using ISH and then sequenced EEEV genome sequences from distinct brain compartments from six patients spanning a wide temporal range from 1938 to 2020. We were able to construct complete or near-complete consensus sequences from 15 samples and > 75% coverage from an additional three samples, allowing the most comprehensive genomic characterization of human EEEV infection to date. Maximum likelihood phylogenetic analysis demonstrated that the sequences from this study clustered with other sequences from similar years and locations; no features unique to human infection were noted. Within patients, consensus sequences from distinct brain compartments contained SNPs predominantly in the nonstructural genes, whereas minority variants were found predominantly in the structural genes.

Our EEEV sequences from humans span a substantial range of time, from one of the first human patients described in 1938 to a recent case in 2020. These add to the few human sequences available in public databases. At the time of this writing, out of 11 EEEV sequences sourced from humans in NCBI virus, two are partial sequences, an additional full-length sequence lacked location data, and the others spanned collection dates from 1947 to 2019. Of these, only two are confirmed directly from patient tissue,^18^ one is confirmed to have been passaged once in Vero cells,^32^ and for the others the passage history, and therefore the effects of cell culture or mouse brain passage, are unclear. Thus, our sequences provide important historical context for EEEV diversity and evolution without the sequence uncertainty introduced through passaging in cells and animals, which is typical of historical isolates.

Our phylogenetic analysis supports prior observations that Florida serves as an EEEV reservoir. Tan et al.^11^ found that EEEV sequences from Florida were diverse and contained clusters of sequences from other areas, suggesting intermittent seeding from a central source. Heberlein-Larson et al.^16^ further identified the Florida Panhandle as the likely source, and two studies identified patterns of population expansion of EEEV in the Northeast congruent with separate introductions from Florida populations.^10^^,^^11^ Our phylogenetic analysis is consistent with multiple EEEV introductions to Massachusetts and further shows that human-derived EEEV sequences cluster with contemporaneous sequences from other hosts.

Sequencing multiple samples per patient allowed detailed analysis of within-host viral diversity throughout the CNS, including the frontal/temporal lobe (cerebral cortex and subcortical white matter), thalamus (deep grey matter), cerebellum, midbrain (brain stem), and spinal cord. Among four patients with multiple samples analyzed, we only identified two consensus-level SNPs. Among two patients with high-depth sequencing of two tissues, we identified a small number of rigorously verified iSNVs. Overall, our results demonstrate low diversity of EEEV in the human CNS. This is similar to another study by our group, which analyzed the neurotropic flavivirus Powassan virus from human brain samples and found no evidence of within-host consensus-level changes in the brain and little variation at the minority level.^33^ Interestingly, a recent study comparing EEEV populations in the blood and CSF from one patient found two consensus-level SNPs between compartments and greater iSNV diversity in the blood than in the CSF, suggesting a potential bottleneck upon the virus entering the CNS.^18^ There was no overlap in either consensus-level or minority variants between our two studies.

Our observation of limited EEEV diversity in the CNS would be compatible with a potential CNS bottleneck, although we did not examine peripheral tissues. We did observe distinct iSNVs in different brain tissues from two patients, suggesting potential compartmentalization. This could arise from separate introductions to distinct brain regions because EEEV is hypothesized to access the brain via a vascular route based on a mouse model.^34^ There could also be cellular factors contributing to compartmentalized replication because different regions of the brain vary in their cell type composition^35^ and protein expression profiles.^36^^,^^37^ The iSNVs we identified may also have arisen de novo as a result of population expansion in the setting of high levels of viral replication in the brain. In the cynomolgus macaque model, after intranasal infection^38^^–^^43^ EEEV typically does not cause detectable levels of viremia; however, multiple studies have found large amounts of viral RNA and replicating virus in the brain,^39^^,^^41^ reportedly ranging up to 10^9^ plaque forming units per gram of tissue.^41^

Eastern equine encephalitis virus ISH staining was able to detect viral RNA in brain sections because all tissues with detectable stain also had EEEV reads identified by sequencing. Stain quantification roughly correlated with EEEV reads per million when > 2% of the tissue area stained. Interestingly, however, regions with the highest EEEV reads per million varied from patient to patient and did not always correspond with stain quantification, possibly due to non-linearity or saturation of ISH intensity within pixels. In the macaque model, despite robust (though unquantified) viral RNA staining in brain sections, staining for viral proteins revealed varying levels of EEEV protein expression with a relatively large amount in the thalamus.^43^ This is in line with observations from previous human tissue antigen staining, which has shown the highest level of EEEV antigen in the thalamus, frontal cortex, and temporal cortex.^8^ In fact, the regions of highest ISH staining and sequence detection for our Patients B and C aligned with the regions of greatest inflammation on magnetic resonance imaging and neuropathology in the original study describing those patients, where they are listed as Patients 10 and 12, respectively.^8^ More broadly, our EEEV ISH staining generally showed the highest amounts of RNA staining in the frontal lobe and thalamus for most patients. However, all ISH and mNGS quantitative data should be interpreted with caution because there is a potential for variability within each of the regions sampled and between serial sections of the same FFPE tissue blocks.

Limitations of our study include the use of FFPE-derived RNA, which can be fragmented, degraded, and difficult to sequence. To account for this, we extensively verified both consensus-level SNPs and iSNVs through visual inspection of reads and verification of nucleotide distribution at SNP locations in mapped read pileup files. Further investigation of EEEV diversity and adaptation in mammalian infection would benefit from additional studies of human CSF and fresh or frozen brain tissue, as well as animal models.

Overall, our study adds important information to the growing field of EEEV molecular epidemiology and pathogenesis by contributing historical and contemporary EEEV consensus sequences from human infection and by demonstrating minimal within-host viral diversity.

## Supplemental Material


Supplemental materials
