# Supplementary material for: Eastern Equine Encephalitis Virus Diversity in Massachusetts Patients, 1938–2020
Source: Am J Trop Med Hyg. 2023 Jun 20;109(2):387–96. doi: 10.4269/ajtmh.23-0047 (PMC10397450; doi:10.4269/ajtmh.23-0047)
Supplement: Supplementary file 1 [file tpmd230047.SD1.pdf]

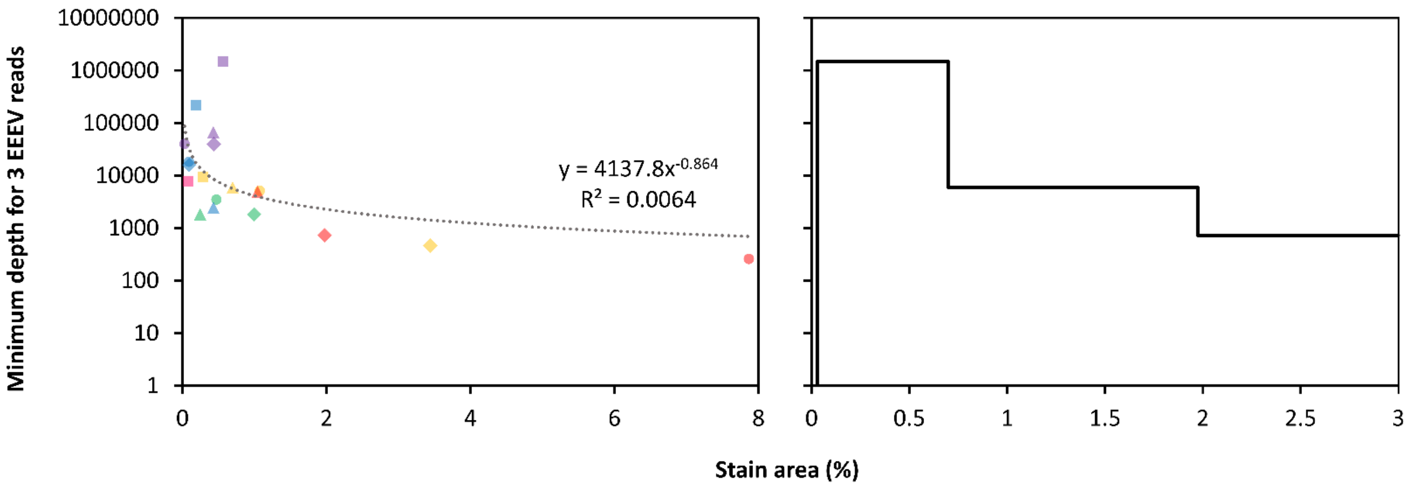

Fig. S1. ***In situ hybridization*** **(ISH) stain quantification required to detect minimum Eastern equine encephalitis virus (EEEV) reads in samples.** EEEV RNA was stained in slides made from brain tissue of EEEV-infected patients, and then stain was quantified and normalized to percent tissue area stained; tissue from the same blocks was then sequenced, and number of EEEV-mapping reads quantified and normalized to number of reads mapped/million. The minimum number of total reads required to reach 3 EEEV reads was calculated for each sample (shaped by patient, colored by brain region) and compared to percent tissue area stained (**A**). Stain thresholds were then calculated to establish minimum required reads to reach 3 EEEV reads for samples within a range of stain quantification.

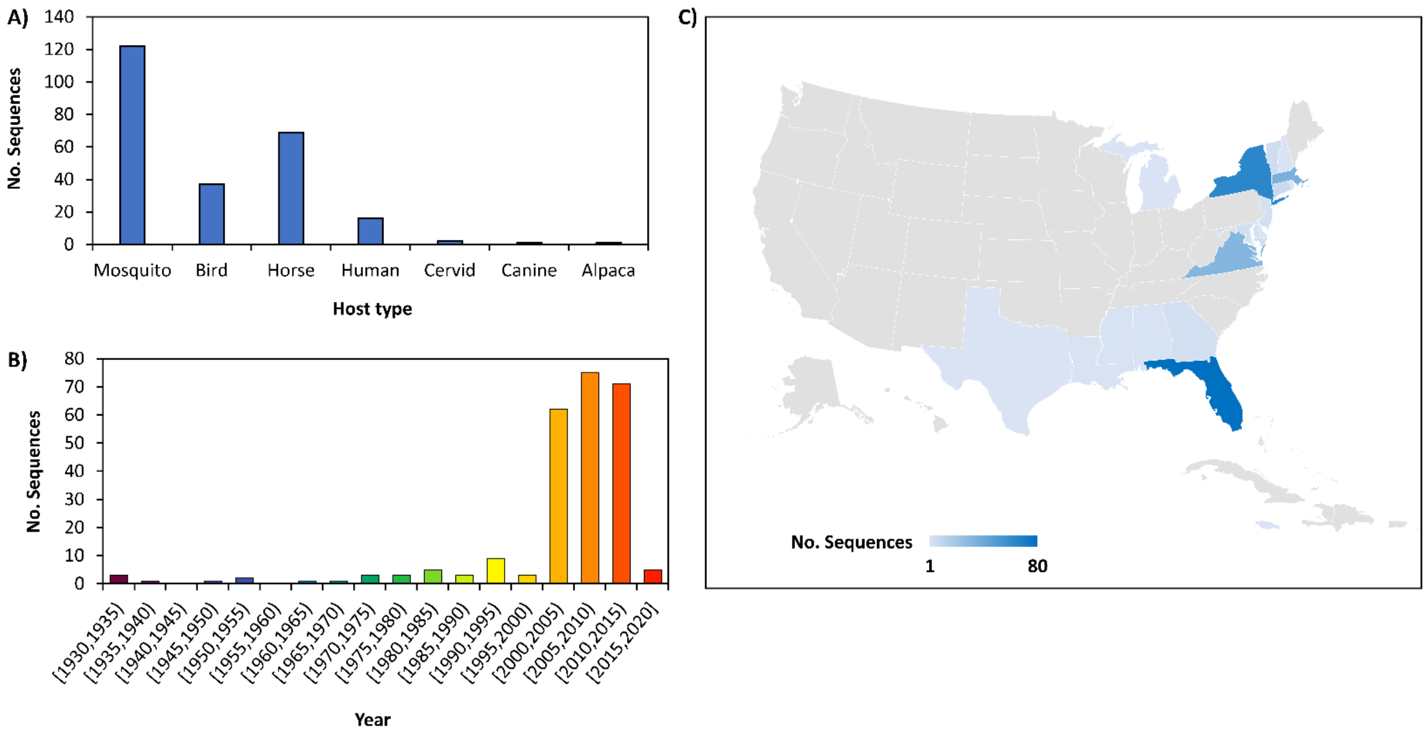

Fig. S2 Temporal, geographic, and host data for complete EEEV genomes. Complete EEEV genomes were downloaded from NCBI virus (https://www.ncbi.nlm.nih.gov/labs/virus/vssi/#/) along with metadata, and then filtered for: complete records of year, host, and location; limited number of "N"s; and identity to other sequences in list. After filtering, metadata was compiled, sequence distribution host **(A)**, by year **(B)**, and location **(C)** were calculated to determine sequence diversity in subsequent analyses.
